# The Clinical Impact of Hexanic Extract of *Serenoa repens* in Men with Prostatic Inflammation: A Post Hoc Analysis of a Randomized Biopsy Study

**DOI:** 10.3390/jcm9040957

**Published:** 2020-03-30

**Authors:** Michael Samarinas, Anastasios Karatzas, Vasileios Tzortzis, Stavros Gravas

**Affiliations:** 1 Department of Urology, Faculty of Medicine, School of Health Sciences, University of Thessaly, Mezourlo, 41110 Larissa, Greece; mikesamih@hotmail.com (M.S.); adkaratzas@yahoo.gr (A.K.); tzorvas@otenet.gr (V.T.); 2 Department of Urology, General Hospital of Larissa, Tsakalof 1, 41221 Larissa, Greece

**Keywords:** hexanic *Serenoa repens*, International Prostate Symptoms Score, maximum flow rate, prostatic inflammation, metabolic syndrome

## Abstract

A randomized biopsy study showed that hexanic *Serenoa repens* (HESr) treatment resulted in prostatic inflammation reduction. This post-hoc analysis evaluated the clinical impact of HESr and investigated correlations between baseline parameters and treatment response. Patients were randomized to receive HESr 320mg/day for six months or no therapy. Assessment included International Prostate Symptoms Score (IPSS), prostate volume (PV), and maximum flow rate (Qmax). Baseline characteristics were recorded, including body mass index (BMI) and metabolic syndrome (MetS) components. In patients under α1-adrenoceptor antagonists (α1-blockers), the addition of HESr resulted in statistically significant IPSS improvement after 6 months (*p* = 0.006). IPSS remained stable in patients under a1-blockers only (*p* = 0.346). Patients treated only with HESr reported a significant IPSS amelioration (*p* = 0.001). In the control group of naïve patients, no significant IPSS change was detected (*p* = 0.298). Baseline PV showed fair correlation (*r* = −0.20) with inflammation reduction in the HESr patients. BMI (*r* = 0.40), diabetes mellitus (*r* = 0.40), and PV (*r* = 0.23) showed fair correlation with Qmax increase but without reaching statistical significance. MetS (*p* = 0.06) was an influent biomarker for Qmax improvement. Treatment with HESr (as monotherapy or add-on therapy to a-blockers) may improve urinary symptoms in terms of IPSS in patients with prostatic inflammation.

## 1. Introduction

Lower urinary tract symptoms (LUTS) have multifactorial etiology, and several urological and non-urological conditions contribute to the development of LUTS [1]. Prostatic inflammation seems to play a role in LUTS pathophysiology, since several studies have reported a relationship between the presence of prostatic inflammation, the severity of LUTS and progression of benign prostatic hyperplasia (BPH). In addition prostatic inflammation has been a common histopathological finding in men treated with prostatectomy [2,3,4]. Prostatic inflammation has also been suggested as a candidate link between metabolic syndrome (MetS) and BPH that could explain the association between these two conditions [5]. Therefore, it was reasonable to explore if therapies that could improve prostatic inflammation might also result in a beneficial effect on patients in terms of alleviation of their urinary symptoms. In this context, different drugs classes with potential anti-inflammatory properties were evaluated with varying results. There is also a growing interest in the use of natural products in treatment of human pathologies, and studies have shown that plant components may exert antiaging, anticancer, anti-inflammatory, and antioxidant activities through different pathways [6,7].

The hexanic lipidosterolic extract of *Serenoa repens* (HESr) is one of the best studied medicinal products both in preclinical and clinical trials [4,8]. HESr acts at different sites in the arachidonic acid cascade by inhibiting prostaglandin synthesis and the production of 5-lipoxygenase metabolites of arachidonic acid. HESr may alter inflammation status by increasing the expression of anti-inflammatory genes and decreasing infiltrates of B lymphocytes, IL-1b, and TNF-a, and the expression of proinflammatory genes [4,8]. Recently, a randomized biopsy study on the effect of HESr on prostatic inflammation was published and showed that treatment with HESr resulted in prostatic inflammation reduction both histologically and immunohistochemically [9].

Here, we present the post-hoc analysis of this randomized biopsy study in order to evaluate the clinical impact of HESr on patients with LUTS and confirmed prostatic inflammation and to identify potential baseline parameters that may be associated with response to treatment.

## 2. Materials and Methods

This randomized blinded trial included patients with confirmed prostatic inflammation after a transrectal prostate biopsy due to increased prostate-specific antigen (PSA) levels and/or a positive direct rectal examination. The concept and the design of the study were previously described in detail [9]. Briefly, eligible patients were randomized into two groups: Group A, who received hexanic *Serenoa repens* 320 mg per day for six months, and Group B, who were given no therapy. Patients were re-evaluated after six months, and a second biopsy was performed according to standard clinical indications.

Assessment at baseline and at six months included the International Prostate Symptoms Score (IPSS), measurement of prostate volume (PV) using transabdominal ultrasound, and maximum flow rate (Qmax). Baseline characteristics were recorded, including body mass index (BMI) and components of MetS such as waist circumference, presence or treatment of hypertension, dyslipidemia and diabetes mellitus. Patients with three of the following five components—central obesity (waist circumference of >102 cm), elevated triglycerides (≥1.7 mmol/L or 150 mg/dL), elevated blood pressure (≥130/85 mmHg), elevated fasting glucose (≥6.1 mmol/L or 110 mg/dL), and reduced HDL cholesterol (<1.03 mmol/L or 40 mg/dL)—were considered to have MetS [10]. Patients with known diabetes mellitus and/or hypercholesteremia and/or arterial hypertension under treatment were also classified as positive for the specific component.

The Wilcoxon test for paired samples (a non-parametric test) was used to compare IPSS and Qmax at baseline and end of treatment for each patient, and *p* value lower than 0.05 was considered to define statistical significance. A Spearman correlation analysis on all available values and a regression model analysis with a backward selection of influent biomarkers were performed to investigate potential correlation between baseline parameters and treatment response. For the interpretation of the Spearman’s correlation coefficients and the report of the correlation strength, Chan’s analysis was used [11].

Statistical analysis was conducted with the use of SPSS Statistics for Windows, v21.0 (IBM Corp, Armonk, NY, USA). The study was approved by the Ethical Committee of the University Hospital of Larissa and registered in the Australian and New Zealand Clinical Trial Registry (ID: ACTRN12613000888763). Informed consent was obtained from all individual participants included in the study.

## 3. Results

Overall, 110 men participated in and completed the study; finally, 97 of them were eligible for the statistical analysis, as thirteen patients were excluded due to the presence of prostate cancer at the second biopsy according to the study design. The HESr group included 49 patients who had received active treatment (age range 56–77 years, mean 71.4), while the control group had 48 patients (age range 58–74 years, mean 68.7). All of them completed the IPSS score (baseline and end of study), but a valuable Qmax (measured at baseline and six months with a voided volume over 150 mL followed by a normal desire for micturition) was finally obtained only in 49 patients, 20 from the HESr group and 29 from the control group. There was no difference in the baseline characteristics between the two groups, as shown previously.

Our study population included patients without any previous treatment for LUTS and patients under treatment with α1-adrenoceptor antagonists (α1-blockers) at baseline. Therefore, a separate analysis was conducted to investigate the impact of HESr on these different sub-populations. In the subgroup of patients treated only with HESr, the IPSS improvement between baseline, and the end of the treatment was statistically significant (*p* = 0.001). On the contrary, in the control group of the naïve patients, there was no statistically significant change in IPSS (*p* = 0.298). Detailed results are presented in Table 1.

Although some patients in the HESr group experienced a significant increase in Qmax, the overall mean Qmax remained practically unchanged—from 11.5 mL/s at baseline to 11.2 mL/s at six months (*p* = 0.7320). The control untreated patients experienced a slight deterioration from 11.4 mL/s to 9.9 mL/s (*p* = 0.096).

The analysis showed that, in patients under a1-blockers, the add-on treatment of HESr resulted in a statistically significant improvement of IPSS (*p* = 0.006). As it was expected, in the control group of patients treated only with a1-blockers, IPSS remained stable (*p* = 0.346). Results are displayed in Table 2.

However, no statistically significant difference in Qmax was found in both subgroups of patients. Mean Qmax of patients under combination therapy with a1-blocker and HESr was 10.5 ml/s and 10.8 ml/s at baseline and end of study, respectively (*p* = 0.317). Control patients who remained under a1-blockers during the study had a mean Qmax of 10.4 ml/s and 10.6 ml/s at baseline and at six months (*p* = 0.510).

Available baseline characteristics such as age, BMI, MetS, and components of MetS were investigated to identify a correlation between those parameters and response to treatment in terms of inflammation reduction, IPSS, and Qmax. Overall, 32 patients (32.9%) met the criteria for MetS, and 16 of them had been treated with HESr. Baseline prostate volume (*r* = −0.20, *n* = 34, *p* = 0.25) showed fair correlation with the response of inflammation in patients treated with HESr. Interestingly, BMI (*r* = 0.40, *n* = 16, *p* = 0.12), diabetes mellitus (*r* = 0.40, *n* = 17, *p* = 0.11), and prostate volume (*r* = 0.23, *n* = 12, *p* = 0.46) showed fair correlation with Qmax improvement. All the other investigated parameters presented poor or no correlation with the outcomes. MetS (*p* = 0.06) was detected as an influent biomarker in the regression analysis for Qmax increase.

## 4. Discussion

The randomized blinded trial on the effect of HESr on prostatic inflammation is the only trial in humans with two prostatic biopsies on the same patient (at baseline and end of study). It was found that treatment with HESr reduced both extension and aggressiveness of inflammation [9].

The present post hoc analysis showed that treatment with HESr 320 mg daily results in a significant improvement in IPSS after six months. The mean IPSS reduction after six months in treatment naïve patients was −3.4 (from 19.8 to 16.4), indicating not only a statistically significant but also a clinically significant improvement. In patients under a1-blockers, the addition of HESr resulted in a further significant improvement of symptoms (from baseline IPSS of 13.3 to the end of study IPSS of 11.3). These results are in line with recently published studies on the efficacy of HESr in men with LUTS. A systematic review analyzed data from 12 Randomized Controlled Trials (RCTs) on the efficacy and safety of HESr [12]. It was concluded that HESr was superior to placebo in terms of improvement of nocturia and Qmax in patients with enlarged prostates. It also showed that the achieved LUTS amelioration was similar to tamsulosin and short-term use of finasteride. An updated systematic review analyzed the available RCTs and also included observational studies. It confirmed the results of the previous systematic review on the efficacy of HESr [13]. A recent network meta-analysis tried to compare the clinical efficacy of *Serenoa repens* (HESr and non-HESr) against placebo and a1-blockers in men with LUTS. Interestingly, only two RCTs on HESr were included in the analysis. It was found that *Serenoa repens* achieved no clinically meaningful improvement against placebo or a1-blockers in short-term follow-up. However, *Serenoa repens* showed a clinical benefit after a long period of treatment, and HESr demonstrated a greater improvement than non-HESr in terms of IPSS [14].

The Quality of Life in Benign Prostatic Hyperplasia (QUALIPROST) study was a longitudinal, prospective, observational, multicenter study aiming at recording the current therapeutic strategies for the management of moderate-to-severe LUTS/BPH in real life and evaluating the changes in quality of life (QoL) and IPSS of patients treated with these therapies [15]. Patients were managed with watchful waiting (WW), monotherapy (a1-blockers, 5aRIs, or phytotherapy), and combination (a1-blocker and 5aRI or a-blocker and HESr). In the phytotherapy group, 95.2% of the patients were offered HESr. At 6 months, improvements in QoL and IPPS in all three medical treatment groups were larger than those recorded in the WW group, and all differences were statistically significant (*p* < 0.05). HESr showed a comparable efficacy to a1-blocker or 5aRI and, similarly, no statistically significant difference was found between combinations of a1-blocker with HESr and a1-blocker with 5aRI [15].

A cross-sectional study compared the combination of HESr with silodosin to silodosin as monotherapy in patients treated for at least for 12 months (mean duration 13.5 months) [16]. It was reported that 69.9% of the combination therapy patients achieved the predefined clinically meaningful improvement (improvement more than three points in baseline IPSS) compared to 30.1% of patients treated only with silodosin (*p* = 0.001). In addition, a higher than 25% improvement in IPSS was found in 68.8% and 31.2% of the patients in the combination and the monotherapy groups, respectively (*p* < 0.001). These data suggest that combination of a1-blocker with HESr results in greater clinically meaningful improvements in LUTS compared to monotherapy with an a-blocker [16].

The anti-inflammatory properties of HESr on prostate have been well proven in vitro and in vivo. Therefore, it would be of clinical interest if these findings could be translated into clinical practice in order to identify patients with prostatic inflammation and treatment responders. Available evidence suggests that MetS and inflammation are important elements in men with LUTS related to BPH and that MetS is associated with larger prostate size [5,17,18]. Our post hoc analysis evaluated several baseline parameters, including MetS components that could be used to predict response to therapy with HESr in men with LUTS. A correlation was found for prostate volume with reduction of inflammation in patients treated with HESr (fair). Interestingly, although overall there was no significant increase in Qmax in the cohort of patients treated with HESr, higher BMI, larger prostate volume, and presence of diabetes mellitus had a fair correlation with Qmax improvement. It should be underlined that *p* values in the correlation analysis were greater than 0.05, possibly due to the small number of patients. However, these findings show a trend toward a better treatment response in patients with specific characteristics. The regression analysis found that MetS was an influent predictor for Qmax improvement.

The main limitation of our study is that, by design, it included only patients with confirmed prostatic inflammation after a prostatic biopsy with elevated serum PSA and/or positive rectal examination, and results should be interpreted with caution. In addition, this is a post-hoc analysis of a study that was powered to investigate if there was a change in prostatic inflammation by the use of HESr. As a result, only few additional parameters could be analyzed. On the other hand, despite the small population, our results confirm the beneficial role of HESr on male LUTS improvement either as monotherapy or as an add-on treatment. Additionally, our study demonstrates a possible positive effectiveness of HESr on patients with LUTS and a specific clinical profile, such as higher BMI, presence of MetS diabetes mellitus, and large prostates. This also underlines that the goal of future research should focus on personalized treatment of LUTS to identify the subgroups of patients who will respond favorably to specific treatments.

## 5. Conclusions

The present study showed that a 6 month treatment with HESr (as monotherapy or as add-on therapy to a-blockers) may improve lower urinary tract symptoms in terms of IPSS in patients with prostatic inflammation.

## Figures and Tables

**Table 1:** Effect of HESr on IPSS in men without previous treatment.

Group		*n*	Range	Mean (SD)	*p*
HESr	IPSS1	25	16–22	19.8 (2.3)	0.001
	IPSS2	25	8–20	16.4 (3.2)	
Controls	IPSS1	25	16–22	19.7 (2.3)	0.298
	IPSS2	25	14–22	18.6 (2.8)	

HESr: hexanic lipidosterolic extract of *Serenoa repens*, IPSS: International Prostatic Symptoms Score, IPSS 1: IPSS at baseline, IPSS2: IPSS at 6 months, N: number of patients, SD: standard deviation.

**Table 2:** Effect of HESr on IPSS as add-on therapy in men under a1-blockers.

Group		*n*	Range	Mean (SD)	*p*
HESr + a1-blocker	IPSS1	24	8–20	13.3 (3.9)	0.006
	IPSS2	24	8–22	11.3 (3.3)	
A1-blocker	IPSS1	23	8–20	13.5 (3.8)	0.346
	IPSS2	23	8–22	13.3 (3.5)	

HESr: hexanic lipidosterolic extract of *Serenoa repens*, IPSS: International Prostatic Symptoms Score, IPSS 1: IPSS at baseline, IPSS2: IPSS at 6 months, N: number of patients, SD: standard deviation.
